# Management of Idiopathic Pulmonary Arterial Hypertension in a Patient in Trinidad: A Case Report

**DOI:** 10.7759/cureus.29699

**Published:** 2022-09-28

**Authors:** Nishtha Mohan, Dominic Dalip, Shiva Jaggernauth

**Affiliations:** 1 Internal Medicine, The University of the West Indies, St. Augustine, TTO; 2 Internal Medicine, Leicester Royal Infirmary, Leicester, GBR; 3 Medicine, Seattle Science Foundation, Seattle, USA; 4 Internal Medicine, Southern Medical Clinic, San Fernando, TTO; 5 Pulmonary Medicine, Apley Medical Clinic, San Fernando, TTO; 6 Pulmonary Medicine, Southern Medical Clinic, San Fernando, TTO

**Keywords:** conventional therapy, pah-targeted therapy, remodulin injection, sildenafil, treprostinil, implantable delivery system, primary pulmonary hypertension, idiopathic pulmonary arterial hypertension

## Abstract

Abnormal elevation in pulmonary arterial blood pressure without secondary causes is Idiopathic Pulmonary Arterial Hypertension (IPAH). It is imperative to establish this diagnosis because IPAH often progresses to right heart failure (RHF) and death without treatment. Right heart catheterization is the standard gold test for diagnosing pulmonary hypertension (PH); however, echocardiography is a susceptible sensitive test and the best non-invasive test. The overall management of IPAH involves supportive measures, conventional therapy, and, pending availability, PAH-targeted therapy. Upon review of the literature, there were no published case reports on IPAH in Trinidad and Tobago. We describe a case of IPAH presented at Apley Medical Centre Limited, Trinidad and Tobago, in the West Indies, emphasizing contemporary management, including using the Implantable Delivery Systems (IDS) for Remodulin injection.

## Introduction

Idiopathic Pulmonary Arterial Hypertension (IPAH), formerly primary pulmonary hypertension, is an abnormal elevation in pulmonary arterial blood pressure without secondary causes [1,2]. It is a rare disease that usually presents shortness of breath [3]. In most cases, IPAH often progresses to right heart failure (RHF) and death without treatment [2]. Echocardiography is the most sensitive test as well as the best non-invasive test; however, right heart cardiac catheterization is the standard gold test in pulmonary hypertension (PH) diagnosis [2,4]. The overall management of IPAH involves general measures, conventional therapy, and, pending availability, PAH-targeted therapy. Despite a strategic outline, management should follow an individualized approach. Upon significant clinical deterioration while on background sildenafil monotherapy, our patient traveled abroad for further management due to the unavailability of parenteral prostanoids in Trinidad and Tobago. She opted for the novel Implantable Delivery System (IDS) for Remodulin Injection (treprostinil) instead of the traditional external pump. This newer therapeutic modality which eliminates many potential barriers to adherence associated with an external pump has not been well documented thus far. This highlights the need for further research regarding the IDS for Remodulin Injection and IPAH management because it is a progressive disease for which there is no cure.

Upon review of the literature, there were no published case reports on IPAH in Trinidad and Tobago. We describe a case of IPAH presented at Apley Medical Centre Limited, Trinidad and Tobago, West Indies, emphasizing contemporary management, including using the IDS for Remodulin Injection.

## Case presentation

**Figure 1:**
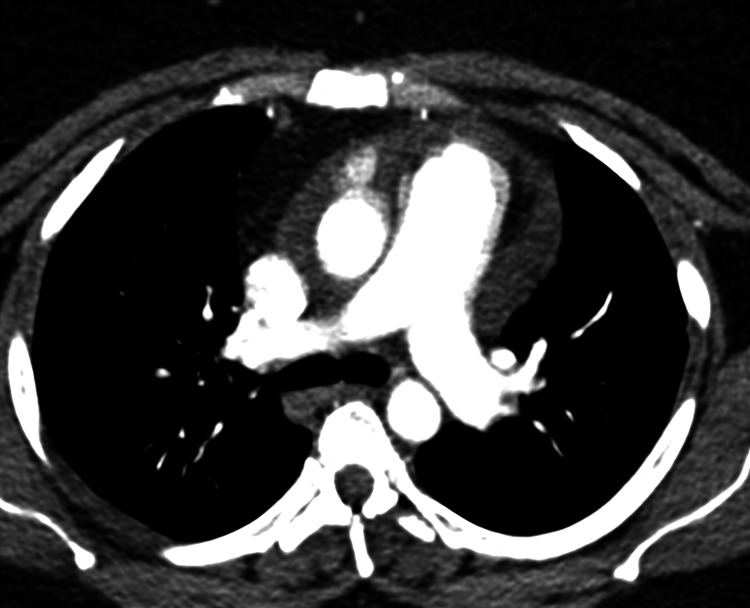
Pulmonary arterial enlargement with no evidence of central emboli

**Figure 2:**
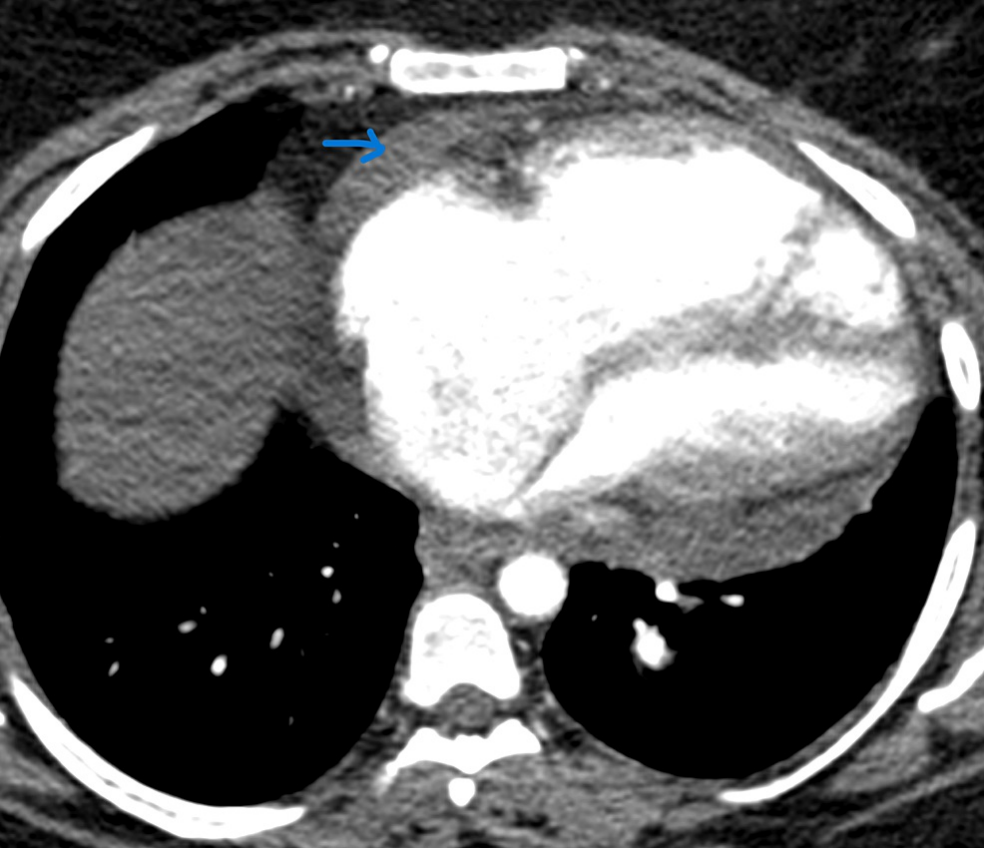
Enlarged right cardiac chambers with small pericardial effusion (Blue Arrow)

A 33-year-old obese female presented to her cardiologist with dyspnea on exertion and pedal edema 12 months ago. She had no evidence of lung disease. Blood investigations revealed hyperlipidemia, high TSH (5.18 uIu/ml), upper limit HbA1c (6.2%), and high CRP (6.07 mg/L). Elevated D-dimer levels (283 ng/ml) prompted CT pulmonary angiogram (CTPA), which ruled out pulmonary thromboembolism (Figure 1). Hematology showed slight leukocytosis, erythrocytosis, and thrombocytopenia; however, further investigations excluded underlying hematological disorders. A Transthoracic Echocardiogram (TTE) was obtained and revealed an elevated systolic pulmonary artery pressure (RVSP) of 86.23 mmHg consistent with PH. Subsequent right heart catheterization (RHC) measured mean pulmonary artery pressure (mPAP) of 108 mmHg, pulmonary capillary wedge pressure (PCWP) of 12 mmHg, and pulmonary vascular resistance (PVR) of 16 Wood units, thereby confirming pulmonary arterial hypertension (PAH). She had no family history of PH, and she denied the use of appetite suppressants and amphetamines. Other TTE findings included dilated right ventricle, right and left atria, right ventricular pressure-volume overload, and moderate pericardial effusion (3.1cm), which was also noted on CT imaging (Figure 2). Normal cardiac biomarkers and no evidence of congenital, valvular or left heart disease on TTE excluded underlying cardiac causes. Given the negative evaluation for associated conditions, she was diagnosed with IPAH, treated with furosemide (20mg po od), spironolactone (12.5mg po od), digoxin (0.125mg po od), sildenafil (12.5mg tid) and apixaban (5mg po od) and referred to a pulmonologist.

After two months, the patient reported 1 episode of vomiting, 1 tsp of blood, and menorrhagia lasting 10 days. The dosage of apixaban was reduced to 2.5mg, and she had a Mirena coil inserted, following which she reported light periods. Her symptoms worsened within the next month with increased dyspnea and pedal edema, associated with 20 lbs weight gain. She complained of anxiety and reduced sleep caused by orthopnea. Polysomnography revealed mild obstructive sleep apnea, and she was started on a CPAP machine which improved her sleep. Repeat TTE showed reduced RVSP from 86.23 to 58 mmHg.

Her condition significantly declined one month later. She was admitted after presenting to the clinic with findings suggestive of worsening RHF - progressive dyspnea, orthopnea by four pillows, reduced exercise tolerance, occasional dizziness, and increased pedal edema. After IV furosemide was administered, her symptoms improved, which lasted two weeks before recurrence. Following the high altitude simulation testing (HAST) procedure which deemed her fit for flight, she traveled to the United States for further investigations. She had an IDS for Remodulin Injection (treprostinil) inserted following the elevation of pulmonary artery pressure to 116 mmHg on a formal pulmonary angiogram. She was also diagnosed with Hashimoto’s thyroiditis by serology testing and treated with levothyroxine.

Although the patient remained limited by dyspnea on exertion (MRC score of 4), her mental status and edema were improved. Four months later, she traveled to the United States, where the IDS for Remodulin Injection was refilled, metolazone 2.5 mg weekly was added, and furosemide was switched to bumetanide 2 mg tid. On her return, she had significantly improved (MRC score of 1) with 20 lbs weight loss; however, she admitted to dizziness. Renal function tests and blood biochemistry were regularly monitored, and supplemental potassium was administered as necessary. Tables 1-5 highlights a summary of results.

**Table 1:** Complete Blood Count laboratory results WBC- White Blood Cells, NEU-Neutrophils, LYM- Lymphocytes, MONO- Monocytes, EOS- Eosinophils, BASO-Basophils, RBC- Red Blood Cells, HBA- Hemoglobin, HCT- Hematocrit, MCV- Mean Corpuscular Volume, MCH- Mean Corpuscular Hemoglobin, MCHC- Mean Corpuscular Hemoglobin Concentration, RDW- Red Cell Distribution Width, PLT- Platelet, MPV- Mean Platelet Volume

Complete Blood Count (CBC)					
Parameters	Reference Range	Units			
			Initial	9-mth	12 mth
WBC	4.0 – 10.5	K/uL	13.57	11.90	10.10
NEU	2.5 – 7.5		7.76	7.90	6.80
LYM	1.5 – 3.5		4.51	3.00	2.40
MONO	0.2 – 0.8		0.90	0.80	0.60
EOS	0.0 – 0.4		0.15	0.20	0.00
BASO	0.0 – 0.2		0.24	0.00	0.00
RBC	3.8 – 5.8	M/uL	6.41	5.27	5.30
HBA	11.5 – 16.5	g/dl	14.28	11.90	11.20
HCT	37 – 47	%	47.34	36.10	35.70
MCV	77– 93	fL	73.84	68.50	66.70
MCH	27– 32	pg	22.27	22.60	21.00
MCHC	32– 36	g/dl	30.16	33.00	31.40
RDW	12.7 – 15.1	%	16.92	18.90	18.40
PLT	150 – 400	K/uL	128.10	237.00	202.00
MPV	0 – 99	fL	9.57	8.60	9.30

**Table 2:** Transthoracic Echocardiogram (TTE) results AO-aortic annulus diameter, LA-left atrium, RV- right ventricle, IVSD-interventricular septum diameter, LVIDD-left ventricular internal dimension at end-diastole, PW- left ventricular posterior wall thickness at end-diastole, LVIDS- left ventricular internal dimension at end-systole, EF-ejection fraction, RVSP- right ventricular systolic pressure, PAEDP-Pulmonary Artery End-Diastolic Pressure

2D Transthoracic Echocardiogram (TTE) M-Mode Measurements						
Parameters	Reference Range	Units				
			Initial	3-mth	9-mth	19-mth
AO	2.0 – 3.1	cm	3.50	3.50	3.10	2.50
LA	2.7 – 3.8	cm	4.30	4.00	4.40	2.90
RV	2.5 – 4.1	cm	4.80	4.30	4.20	1.50
IVSD	0.6 – 0.9	cm	1.40	0.90	0.90	0.80
LVIDD	3.8 – 5.2	cm	2.10	3.10	3.40	4.50
PW	0.6 – 0.9	cm	1.20	0.90	0.70	0.50
LVIDS	2.2 – 3.5	cm	1.30	2.40	1.80	2.80
EF	54 – 74	%	70.00	56.00	70.00	77.00
RVSP	>35	mmHg	86.23	65.43	68.15	25.68
PAEDP	>15	mmHg	-	22.10	-	-

**Table 3:** Right Heart Catheterization results RA-right atrium pressure, PA-pulmonary artery, PCWP-pulmonary capillary wedge pressure, SVC O2 sat- mixed venous oxygen saturation, RA O2 sat- right atrium oxygen saturation, PA O2 sat- pulmonary artery oxygen saturation, AO O2 sat- aorta oxygen saturation, Fick (CO)- Fick's cardiac output, Thermo-dilution (CO)- thermo-dilution cardiac output, Fick (CI), Fick's cardiac index, Thermo-dilution (CI), Thermo-dilution cardiac index, PVR- pulmonary vascular resistance, RVSWI- right ventricular stroke work index, PAPi- pulmonary artery pulsatility index, TPG- transpulmonary gradient, DPG- diastolic pulmonary gradient

Right Heart Catheterization			
Parameters	Ref Range	Units	13-mth Follow up
RA	0 - 5	mmHg	28/21/20
RV	20/5	mmHg	106/3/24
PA	20/10/15	mmHg	108/35/64
PCWP	7 - 12	mmHg	12/10/7
SVC O2sat	60 - 80	%	46.00
RA O2sat	75	%	36.00
PA O2sat	75	%	36.00
AO O2sat	95 - 100	%	90.00
Fick (CO)	4 - 8	L/min	3.50
Thermo – dilution (CO)		L/min	3.60
Fick (CI)	2.8 – 4.2	L/min /m2	1.50
Thermo – dilution (CI)		L/min /m2	1.60
PVR	<3.125	WU	160
RVSWI	8 - 12	g/ m/beat /m2	8.90
PAPi	= 0.9		3.65
mRAP/ PCWP			2.80
TPG	= 12		57.00
DPG	<7		28.00

**Table 4:** Renal Function Tests results ALB- albumin, Glob-globulin, A/G- albumin/globulin, BUN- blood urea nitrogen, Creat- creatinine, Na- sodium, K- potassium, Cl- chloride, EGFR- estimated glomerular filtration rate

Renal Function Tests (RFT)							
Parameters	Reference Range	Units					
			Initial	9-mth	12 mth	17-mth	19-mth
Protein (Total)	65 – 83	g/L	72.00	75.00	71.00		78.40
ALB	35 – 52	g/L	38.00	39.00	40.00		40.30
Glob	23 – 35	g/L	34.00	36.00	31.00		38.10
A/G	1.1 – 2.5		1.10	1.00	1.30		1.00
BUN	7.0 – 18.7	mg/dL	12.20	9.00	11.00	12.00	10.20
Urea	15 – 40	mg/dL	26.00	19.00	23.00		21.00
Creat	0.57 – 1.11	mg/dL	0.96	0.79	0.81	0.90	0.77
Urate	2.6 – 6.0	mg/dL	10.50	4.90	6.90		10.40
Na	136 – 145	mmol/L	138.00	138.00	140.00	137.00	136.00
K	3.5 – 5.1	mmol/L	4.10	4.80	3.60	3.80	3.62
Cl	98 – 107	mmol/L	103	106.00	105.00	100.00	98.00
Iron	50 – 170	ug/dl	26.00	34.00	23.00		29.00
E GFR			67.00	83.00	98.00	85.00	103.00

**Table 5:** Additional Investigations CRP- c reactive protein, HbA1c- glycated hemoglobin, TSH- thyroid stimulating hormone, T3- triiodothyronine, Bili (T1)- total bilirubin, Bili (D)- direct bilirubin, Bili (I)- indirect bilirubin, Alk Phos- alkaline phosphatase, AST- aspartate aminotransferase, ALT- alanine transaminase, BNP-brain natriuretic peptide

Additional Investigations						
Parameters	Reference Range	Units				
			Initial	12 mth	13-mth	17-mth
CRP	0 – 5	mg/l	< 5.00	24.40		
HbA1c	4.5 – 6.3	%	6.20			
D-dimer	0 – 198	ng/ml	283.00			
TSH	0.35 – 4.94	uiu/ml			10.96	
T3	0.58 – 1.59	ng/ml			0.43	
Bili (Tl)	0.2 – 1.2	mg/dl		2.20		
Bili (D)	= 0.2	mg/dl		0.90		
Bili (I)	0.2 – 1.2	mg/dl		1.30		
Alk Phos	31 – 125	U/L		122.00		
AST	10 – 30	U/L		19.00		
ALT	6 – 29	U/L		13.00		
Digoxin	0.8 – 2.4	ng/ml		2.20		
BNP	<100	pg/ml				414

## Discussion

IPAH, belonging to the WHO Group 1 PH, refers to pulmonary arterial hypertension (PAH) without a secondary cause. Formerly known as Primary Pulmonary Hypertension prior to the 5th World Symposia on Pulmonary Hypertension, IPAH is an uncommon, incurable disease with an incidence of 4 to 6 cases per million people worldwide. It is more common in females presenting in their forties [4,5].

According to the REVEAL study, our patient presented with year-long dyspnea on exertion, the most frequent presenting symptom of PAH, placing her into WHO-FC/NYHA functional class II (Table 6). Her physical examination revealed characteristic findings, including pedal edema and tricuspid regurgitation murmur [6,7]. Therefore, clinical suspicion of PH prompted diagnostic investigations.

**Table 6:** WHO functional assessment for pulmonary hypertension (modified from New York Heart Association functional classification)

WHO Functional Class (FC)	Description
I	Patients with pulmonary hypertension without limitation of physical activity. Ordinary physical activity does not cause dyspnea or fatigue, chest pain or near syncope.
II	Patients with pulmonary hypertension with slight limitation of physical activity.
III	Patients with pulmonary hypertension with marked limitation of physical activity.
IV	Patients with pulmonary hypertension with inability to perform any physical activity without symptoms. These patients manifest signs of right heart failure. Dyspnea and/or fatigue are present at rest.

Measurements taken by RHC, which represents the gold standard of PAH diagnosis, in the setting of consistent TTE findings, confirmed our patient’s diagnosis. Evidence of right ventricular hypertrophy on CXR and ECG may also be found in PH patients [4]. Upon detecting PH, Pulmonary Function Tests (PFT), Arterial Blood Gas (ABG), and CT studies are performed to identify the WHO Group to which the patient belongs, followed by blood tests and serology to evaluate which type [4,7,8]. In our patient, CTPA ruled out thromboembolism and reduced lung diffusion capacity for carbon monoxide (DLCO) was indicative of Group 1. Serology ruled out underlying connective tissue disease and hepatitis [4,8]. Furthermore, extensive blood investigations excluded secondary causes, categorizing our patient as IPAH.

Poor prognostic indicators include being over 50 years old, lower body mass index, functional class III or IV, DLCO less than or equivalent to 45% of predicted, and absence of PAH-targeted therapy [4,8,9]. Our patient had a pericardial effusion and evidence of RHF, which also confers a poor prognosis [8,9].

Although PAH management is guided by an outlined strategy coupled with follow-up visits to monitor clinical response, it should be tailored to each patient’s co-morbidities, complications, and preferences [10]. While managing our patient’s IPAH, high HbA1c levels and positive anti-thyroglobulin antibodies were discovered; subsequently, metformin and levothyroxine were added to her regimen. Notably, the development of thyroid disease is common in PAH [8]. Our patient was also found to have mild obstructive sleep apnea; thus, we introduced a CPAP machine to ameliorate her orthopnea. Nocturnal hypoxia, which can both cause and exacerbate PH, has a high prevalence in PAH (70-80%); moreover, CPAP treatment can reduce PH symptoms [8,11].

The current PAH treatment algorithm is divided into three steps according to the Grade of Recommendation and Level of Evidence assigned by the European Society of Cardiology/European Respiratory Society [8]. Moreover, the overall treatment goal is to bring or keep the patient in WHO-FC II when possible [8]. The therapeutic strategy includes an initial approach consisting of general measures, conventional therapy, expert referral, acute vasoreactivity testing; calcium channel blockers (CCBs) in vasoreactive patients or PAH-targeted therapy in non-vasoreactive patients, and response to the initial treatment strategy [8,10]. In surgical management, transplantation is an option for eligible patients with severe disease refractory to maximal medical therapy. Interventional procedures, such as balloon atrial septostomy (BAS), are considered a temporizing measure for patients awaiting transplantation or without access to medical therapy [1,10,12-23]. Our patient adopted the general measures advised by her physician corroborated in the literature, which indicates avoiding strenuous physical activity [8,10]; recommends avoiding pregnancy (associated with a very high maternal mortality rate) [4,8,10]; recommends influenza and pneumococcal vaccinations (pneumonia is the cause of death in 7% of PAH cases) [8,10].

As there were no protocol-mandated PAH treatments in the past, conventional or supportive therapy such as anticoagulants, diuretics, and cardiac glycosides were prescribed as necessary, all of which were included in our patient’s treatment regimen [4,8,9,23-30]. Oxygen therapy would be added if SpO2 <90% [31]. Judicious use of diuretics is crucial to reduce volume overload without consequent hypotension (due to reduced cardiac output as the right heart is preload dependent), arrhythmias (due to hypokalemia), and metabolic alkalosis [23,25,27,29]. As such, renal function and blood biochemistry were regularly monitored when bumetanide and metolazone were added to our patient’s regimen, and she was treated with supplemental potassium as necessary. Studies have shown that acutely administered inotropic drugs, such as digoxin, have improved cardiac output in IPAH; however, its long-term effects remain unknown [25,27,30]. The role of anticoagulation in IPAH is controversial as the evidence in favor was derived from small, retrospective, and single-center studies [25,26]. Nonetheless, in light of our patient’s high BMI and sedentary lifestyle, as well as the theoretical risk for intrapulmonary thrombosis in an already compromised pulmonary vascular bed, she was treated with apixaban. In contrast, the literature recommends warfarin [23,25-28].

During RHC, patients should undergo pulmonary vasoreactivity testing with a vasodilator to determine if they belong to the 10% of patients with IPAH who exhibit a positive acute vasoreactive response and, thus, will likely benefit from CCB monotherapy [32]. The potential danger is hemodynamic collapse, and as such, CCBs are contraindicated in patients with low cardiac output states and elevated right atrial pressure (>20 cm H2O) [12]. Non-vasoreactive patients initiate one or more of the three PAH-targeted drugs (Table 7), which have become the contemporary standard of practice [1]. A Japanese retrospective study attributed the improvement of hemodynamic parameters to using Endothelin Receptor Antagonists (ERA) and IV epoprostanoid [12]. A randomized trial showed similar results when survival in patients on IV epoprostanoid plus conventional therapy was compared with those on conventional therapy alone [13].

**Table 7:** Mechanisms of action and clinical benefit of calcium channel blockers and approved PAH-targeted drugs used in the medical management of Idiopathic Pulmonary Arterial Hypertension IPAH, idiopathic pulmonary arterial hypertension; PVR, pulmonary vascular resistance; PAH, pulmonary arterial hypertension; CTD, connective tissue disease; HIV, human immunodeficiency virus; AS, atrial septostomy; DLTx, double lung transplantation; cGMP, cyclic guanosine monophosphate; mPAP, mean pulmonary artery pressure; NO; nitric oxide

Drug	Mechanism of Action	Clinical Benefit
Calcium Channel Blockers (CCB)		
Nifedipine	Vasodilation against smooth muscle cell hypertrophy, hyperplasia and vasoconstriction seen in patients with IPAH	Improvement in functional class of I or II and hemodynamics
Diltiazem		
Amlodipine		
Prostanoids/ Prostacyclin Analogues		
Epoprostenol	Promotion of direct arterial vasodilation and inhibition of platelet aggregation	Improvement in survival in IPAH [Barst] symptoms, exercise capacity and hemodynamics
Treprostinil		Improvement in symptoms and exercise capacity
Iloprost		Improvement in symptoms, exercise capacity, PVR and clinical events
Endothelin Receptor Antagonists (ERA)		
Bosentan	Blockade of endothelin-1 receptors as endothelin-1 (a potent vasoconstrictor) is overexpressed in the plasma and lung tissue in patients with IPAH	Improvement in functional class, exercise capacity, hemodynamics, echocardiographic and Doppler variables and time to clinical worsening
Ambrisentan		Improvement in symptoms, exercise capacity, hemodynamics and time to clinical worsening in IPAH and PAH associated with CTD, HIV and Eisenmenger’s syndrome
Macitentan		Improvement in exercise capacity and reduction in composite endpoint of death, AS, DLTx, initiation of prostanoids or worsening of PAH
Phosphodiesterase Type-5 Inhibitors (PDE-5i)		
Sildenafil	Prevention of hydrolysis of cGMP which has vasodilatory and antiproliferative effects on pulmonary vasculature	Improvement in functional class and exercise capacity and reduction of mPAP with all doses
Tadalafil		Favorable results on symptoms, exercise capacity, hemodynamics and time to clinical worsening
Soluble Guanylate Cyclase Stimulators (sGC)		
Riociguat	Dual mode of action, acting in synergy with endogenous NO and also directly stimulating sGC independent of NO availability	Favorable results on functional class, exercise capacity, hemodynamics and time to clinical worsening

Furthermore, PAH-targeted therapies can be administered in combination, sequential or initial, as simultaneously targeting different mechanisms involved yields more favorable outcomes than increasing the dose of a single drug [4]. Sequential combination therapy is the most widely utilized strategy, where a drug(s) is added to monotherapy in the event of inadequate clinical response or deterioration [1,8,10]. Our patient started on monotherapy with sildenafil, FDA approved for PAH treatment in 2005 [1]. While there is no evidence-based monotherapy [8], Phosphodiesterase-5 inhibitors (PDE 5i), the most widely prescribed PAH-targeted therapy, are typically the initial therapy prescribed [1]. Our patient underwent sequential combination therapy where a prostanoid was added to sildenafil after she was admitted for decompensated RHF. Although this contrasts with the most common combination therapy, ERA plus PDE-5i [1,4], the 2008 PACES randomized trial showed improved exercise capacity in patients on IV prostanoid and PDE-5i [1,14]. Notwithstanding, initial or upfront combination therapy has emerged as a widely accepted standard of care [1,10].

Treprostinil, a prostanoid, was added to the management of our patient using a fully implantable, programmable intravascular delivery system (IDS). Procurement of this drug, however, required the patient to take the necessary steps to acquire special authorization from the Government of Trinidad and Tobago. As the first IDS to be FDA approved for PAH therapy (2018), the novel IDS for Remodulin Injection (treprostinil) came as a breakthrough application mode in IPAH management [15,16]. It represents a beneficial alternative to previously used external pumps as it yields a 44-fold reduction of catheter-related severe bloodstream infections and a reduction of catheter thrombosis, occlusions, or kinks, as revealed by the DellVery study [16,17]. Moreover, patients experience a higher quality of life, such as taking showers without concern for getting the pump wet, sleeping without fear of catheter dislocation, reduced frequency of refillings, and reduced stigmatizations associated with the visibility of external pumps [16-18]. However, studies reveal local and systemic treprostinil-related reactions after refilling and cases of pump failure [1,16]. Similar to the participants of the DellVery study, our patient was satisfied with her IDS for intravenous treprostinil [18,19].

The only prostacyclin currently used with the IDS system is treprostinil due to its long-term thermal stability compared to epoprostenol, which is considered the most effective therapy as it is the only PAH-targeted therapy to have shown improved survival in a randomized controlled trial [1,4,16-19]. Notwithstanding, the result of another trial elucidates that continuous subcutaneous treprostinil, in addition to conventional therapy, is effective in IPAH patients as measured by improvements in exercise capacity, hemodynamics and symptoms when compared to those treated with conventional therapy alone [1,20]. Additionally, the 2010 TRIUMPH I trial in which 30% of participants had background sildenafil showed a favorable outcome evident by improved exercise capacity [1], which is in keeping with our patient’s results following sequential combination therapy with sildenafil and treprostinil.

Transplantation will be the final viable option if our patient’s condition deteriorates to WHO-FC III or IV symptoms, despite maximal dosage of medical therapy [1,8,10,22,24,33]. Both double lung (DLTx) and heart-lung (HLTx) transplantations have been performed for PAH, with studies showing similar outcomes after DLTx as after HLTx [22,33-35]. While DLTx is the preferred treatment, HLTx is usually reserved for PAH related to congenital heart disease and when there is significant, irreversible right or left heart failure [1,22,34]. Published reports have proposed that creating an interatrial shunt by BAS allows for Eisenmenger physiology which increases left ventricular preload and systemic blood flow, consequently providing symptomatic and hemodynamic improvement [1,10,22,23,36]

The major limitation in our case report is limited therapeutic choices in the management of our patient due to the unavailability of parenteral prostanoids in Trinidad and Tobago, with the only option being inhaled Iloprost.

## Conclusions

In conclusion, we report a case of IPAH in Trinidad, West Indies, that was not previously reported in the literature. After detecting PH, extensive evaluation and detailed history-taking for associated causes should promptly ensue as IPAH is a progressive disease for which there is no cure. Management of our patient consisted of conventional therapy and sequential combination therapy of the PAH-targeted drugs, sildenafil, and treprostinil. Moreover, she opted for the new therapeutic modality, the IDS for Remodulin Injection, after which her symptoms and functional capacity significantly improved.
